# *α*-klotho as a biomarker of amyloid *β* levels in the cerebrospinal fluid

**DOI:** 10.3389/fnagi.2025.1599402

**Published:** 2025-06-23

**Authors:** Jacob Raber, Alexandra Pederson, Emily Bunnell, Nora Mattek, Nora E. Gray, Steven G. Kohama, Alison R. Weiss, Joseph F. Quinn, Henryk F. Urbanski

**Affiliations:** ^1^Department of Behavioral Neuroscience, Oregon Health and Science University, Portland, OR, United States; ^2^Department of Neurology, Oregon Health and Science University, Portland, OR, United States; ^3^Department Radiation Medicine, Oregon Health and Science University, Portland, OR, United States; ^4^Division of Neuroscience, Oregon National Primate Research Center (ONPRC), Oregon Health and Science University, Portland, OR, United States; ^5^Division of Reproductive and Developmental Science, ONPRC, Oregon Health and Science University, Portland, OR, United States

**Keywords:** *α*-klotho, apoE, Aβ, CSF, serum, rhesus macaques, cognitive healthy elderly, dementia

## Abstract

**Introduction:**

CSF *α*-klotho levels might affect Aβ40, Aβ42, and the Aβ42/40 ratio in the cerebrospinal fluid (CSF).

**Methods:**

CSF *α*-klotho was assayed in ovariectomized rhesus macaques (NHPs) maintained on a Western-style diet (WSD) to assess the effect of estrogen hormone therapy (HT). CSF and serum *α*-klotho was also analyzed in females and males of different ages and whether it was associated with Aβ42, Aβ40, or the Aβ42/40 ratio. Furthermore, CSF and serum *α*-klotho were analyzed in women and men with dementia and controls and whether they were associated with CSF Aβ levels.

**Results:**

HT was associated with increased CSF *α*-klotho levels. Furthermore, *α*-klotho and Aβ levels were correlated in a species- and cognitive health-dependent fashion. Higher CSF and serum levels of *α*-klotho were seen in controls than in patients with dementia.

**Discussion:**

Understanding the species differences in the beneficial effects of *α*-klotho on CSF Aβ physiology should open new avenues for treating AD.

## 1 Introduction

It is plausible that *α*-klotho, a geroprotective protein (Kanbay et al., 2023), plays an important role in the risk of developing neurodegenerative diseases (Kuro-O, 2019). In support of this view, heterozygote carriers of the KL-VS klotho variant had two- to three-fold higher serum levels of *α*-klotho than non-carriers and showed better global cognition than non-carriers (Dubal et al., 2014). Additionally, heterozygote carriers of the KL-VS klotho variant showed less amyloid-dependent tau accumulation and less memory impairments in AD (Neitzel et al., 2021). Moreover, *α*-klotho levels in the CSF were lower in individuals with Alzheimer’s disease (AD) than in age-matched controls (Gupta et al., 2022). Also, serum *α*-klotho levels were found to be positively correlated with cognitive status in a cohort of AD patients and controls (Kundu et al., 2022).

*α*-klotho has been shown to increase cognitive performance in aged Nonhuman Primates (NHPs) and to slightly increase cognitive performance in young mice (Castner et al., 2023). In mice, CSF *α*-klotho levels were related to cognition (Kuro-O, 2019; Dubal et al., 2014). Increasing *α*-klotho levels in the brains of mice with transgenic expression of human amyloid precursor protein and presenilin 1 containing AD mutations reduced amyloid pathology and improved cognitive performance (Zhao et al., 2020). In NHPs however, relatively little is known about the relationship between *α*-klotho levels in CSF and serum and CSF levels of Aβ measures.

In the NHP Radiation Survivor Cohort, which received 6.5–8.05 Gy of ionizing radiation, amygdala *α*-klotho and apolipoprotein E levels (apoE) were positively correlated and apoE levels in the amygdala predicted relative hippocampal volume (Kundu et al., 2021). ApoE plays an important role in lipid transport and neuronal repair following injury (Mahley, 1988) and was found to increase in the brains of aged NHPs (Haley et al., 2010) and in mice following environmental challenges such as irradiation (Haley et al., 2012).

NHPs share a high genetic homology with humans (Singh and Olabisi, 2017) and show changes in the levels of biomarkers of AD in the cerebrospinal fluid (CSF), including amyloid Aβ42 which in turn is associated with hippocampal and cortical atrophy and cognitive impairments (Darusman et al., 2014), but they do not develop dementia. In a NHP model of menopause and estrogen hormone therapy (HT) (Kohama and Urbanski, 2024) on a regular and a Western-style diet (WSD), HT was associated with reduced amyloid *β* (Aβ) pathology in the amygdala (Appleman et al., 2024). In the present study, we investigated whether in the CSF of ovariectomized (OVX) rhesus macaques (NHPs) on a WSD, *α*-klotho levels are increased by HT, whether in these and other NHPs *α*-klotho levels in CSF and serum are correlated with CSF Aβ measures, and whether there are sex differences in these relationships. In addition, we assessed whether in women and men with and without dementia *α*-klotho levels in CSF and serum are correlated with CSF Aβ measures.

## 2 Materials and methods

### 2.1 HT

HT in old (19–26 years) OVX NHPs maintained on a WSD for about 30 months is described in Prokai et al. (2023). Briefly, 11 NHPs were ovary-intact controls and remained on a chow diet (Monkey Diet, LabDiet, Inc., St Louis, MO, USA) and 22 were OVX and exposed for ∼30 months to a WSD (TAD Primate Diet; LabDiet, Inc.), which provided calories with 36% fat, 44% carbohydrates (includes 18.5% sugars) and 18% protein. Regular monkey chow provides calories with 13% fat, 69% complex carbohydrates (includes 6% sugars) and 18% protein. Of the OVX NHPs, 8 received empty subcutaneous capsules, 7 received HT by estradiol (E2) in subcutaneous elastomer capsules beginning immediately after OVX and 7 received delayed HT for 6 months (Purnell et al., 2019). The E2 capsules were designed to maintain serum E2 concentrations between 100 and 200 pg/mL, which is similar to concentrations during the mid-to-late follicular phase of the menstrual cycle (Bethea et al., 2016).

### 2.2 All NHPs

CSF was analyzed in 102 NHPs: 8 female and 13 male young-adults (4–9 years), 15 female and 14 male middle-aged (10–18 years), 29 female and 20 male old (19–26 years), and 3 female oldest-old (27 years and older). Eight of the old males received testosterone and DHEA supplementation for 6 months, as described Urbanski (2017). Serum was analyzed in 49 of the 102 NHPs, using all of the archived samples we had available in our freezers: 1 female and 12 male young-adults (4–9 years), 8 female and 12 male middle-aged (10–18 years), 7 female and 7 male old (19–26 years), and 2 female oldest-old (27 years and older). Four of the old males had received testosterone and DHEA supplementation for 6 months.

### 2.3 NHP CSF

CSF was collected at necropsy from rhesus macaques that were involved in various unrelated studies. The samples were collected from the cisterna magna.

### 2.4 Humans

The human CSF and serum samples were banked specimens from the OHSU Layton Aging and Alzheimer’s Center, as described (Kundu et al., 2022). CSF was analyzed in 94 humans. The age range was 39–83 + years of age. All subjects with a banked CSF sample, regardless of diagnosis, were included. There were 26 female and 15 male controls and 19 female and 34 male dementia patients. Of the demented patients, 19 males and 9 females were diagnosed with probable AD, 5 males and 1 female with possible AD, 6 males and 4 females with questionable dementia, 1 male and 2 females with other dementias, 1 male with frontotemporal dementia, and 2 males and 1 female with asymptomatic familial AD.

### 2.5 *α*-klotho ELISA

The analysis of *α*-klotho in the NHP samples was performed using human *α*-klotho ELISAs from Immuno-Biologic Laboratories (Code number 27998, lot number 2H-406), Minneapolis, MN. All NHP samples were diluted 1:3 in diluent. *α*-klotho levels in the human CSF and serum samples were analyzed as previously reported (Kundu et al., 2022).

### 2.6 apoE ELISA

The analysis of apoE in the NHP CSF samples was performed using human apoE ELISAs from Sigma (RAB0613-1KT, Lot number 0414J61, Saint Louis, MO). All NHP samples were diluted 1:25 in diluent. ApoE levels in the human serum and CSF samples were analyzed previously as described (Kundu et al., 2022).

### 2.7 Aβ ELISAs

Aβ40 and Aβ42 in the human CSF samples was analyzed using MyBioSource- ELISAs (Aβ42; catalog number: MBS268504, Lot number: 38475195; Aβ40: catalog number: MBS760432, lot number: FN250109), San Diego, CA. All human CSF samples were diluted 1:10 in diluent. NHP Aβ40 and Aβ42 was analyzed by Myriad RBM (Austin, TX, USA) using Luminex technology and Human CustomMAP (HMPC109). The least detectable doses were 0.018 and 0.055 ng/mL, respectively.

The ELISAs were performed according to the manufacturer’s instructions. Test dilutions were used to ensure that *α*-klotho, apoE, and Aβ40, and Aβ42 levels fell within the standard curve range. Absorbances were read using an ID5reader (Molecular Devices, San Jose, CA).

### 2.8 Statistical analyses

All statistical analyses and figures were generated using GraphPad Prism software (version 10.4.1, San Diego, CA). To assess effects of HT on CSF *α*-klotho and apoE levels, one-way ANOVAs with a Dunnett’s *post-hoc* tests were used. To assess relationships between *α*-klotho and, Aβ40, Aβ42, the Aβ42/40 ratio, and apoE correlational analyses were performed. To determine whether CSF and serum *α*-klotho levels differed between demented patients and controls, 2-tailed unpaired Student *t*-tests were used.

## 3 Results

### 3.1 HT NHP study

**Figure 1:**
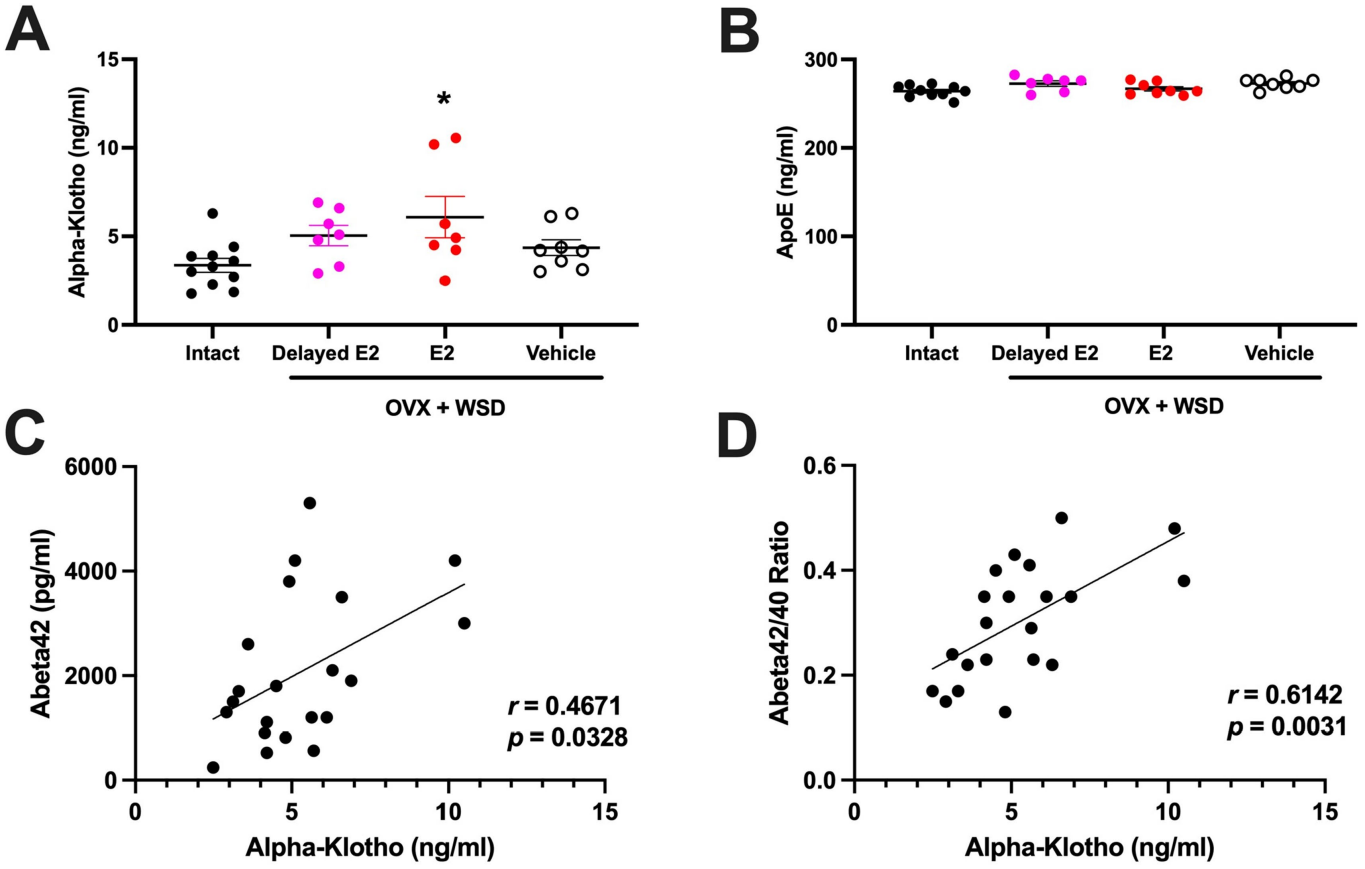
**(A)** There was an effect of estrogen treatment on CSF *α*-klotho levels. Higher levels were observed in OVX animals that were maintained on a Western-style diet and subjected to estradiol replacement therapy immediately after OVX, when compared to the levels in ovary-intact animals. *p < 0.05 (Dunnett’s). **(B)** There was no effect of estrogen treatment on CSF apoE levels. **(C)** CSF levels of *α*-klotho and Aβ42 were positively correlated (*r* = 0.4671, *p* = 0.0328, Spearman). **(D)** CSF levels of *α*-klotho levels and Aβ42/40 ratio were also positively correlated (*r* = 0.6142, *p* = 0.0031, Spearman). In panels C and D, data of animals are displayed regardless of treatment.

There was an effect of HT on CSF *α*-klotho levels [*F*_(3,29)_ = 3.195, *p* = 0.0381], with higher *α*-klotho levels in OVX animals on a WSD who received estradiol (E2) immediately after OVX than in ovary-intact controls (*p* = 0.0152, Dunnett’s) (Figure 1A). In contrast, there was no effect of HT on CSF apoE levels (Figure 1B). The CSF *α*-klotho and Aβ42 levels were positively correlated (Figure 1C). The CSF *α*-klotho levels and Aβ42/40 ratio were also positively correlated (Figure 1D). In contrast, there was no significant correlation between CSF *α*-klotho and Aβ40 levels (*r* = 0.1527, *p* = 0.5087). Similarly, there were no significant correlations between CSF apoE levels and Aβ40 levels, Aβ42 levels, nor the Aβ42/40 ratio.

### 3.2 All NHPs

**Figure 2:**
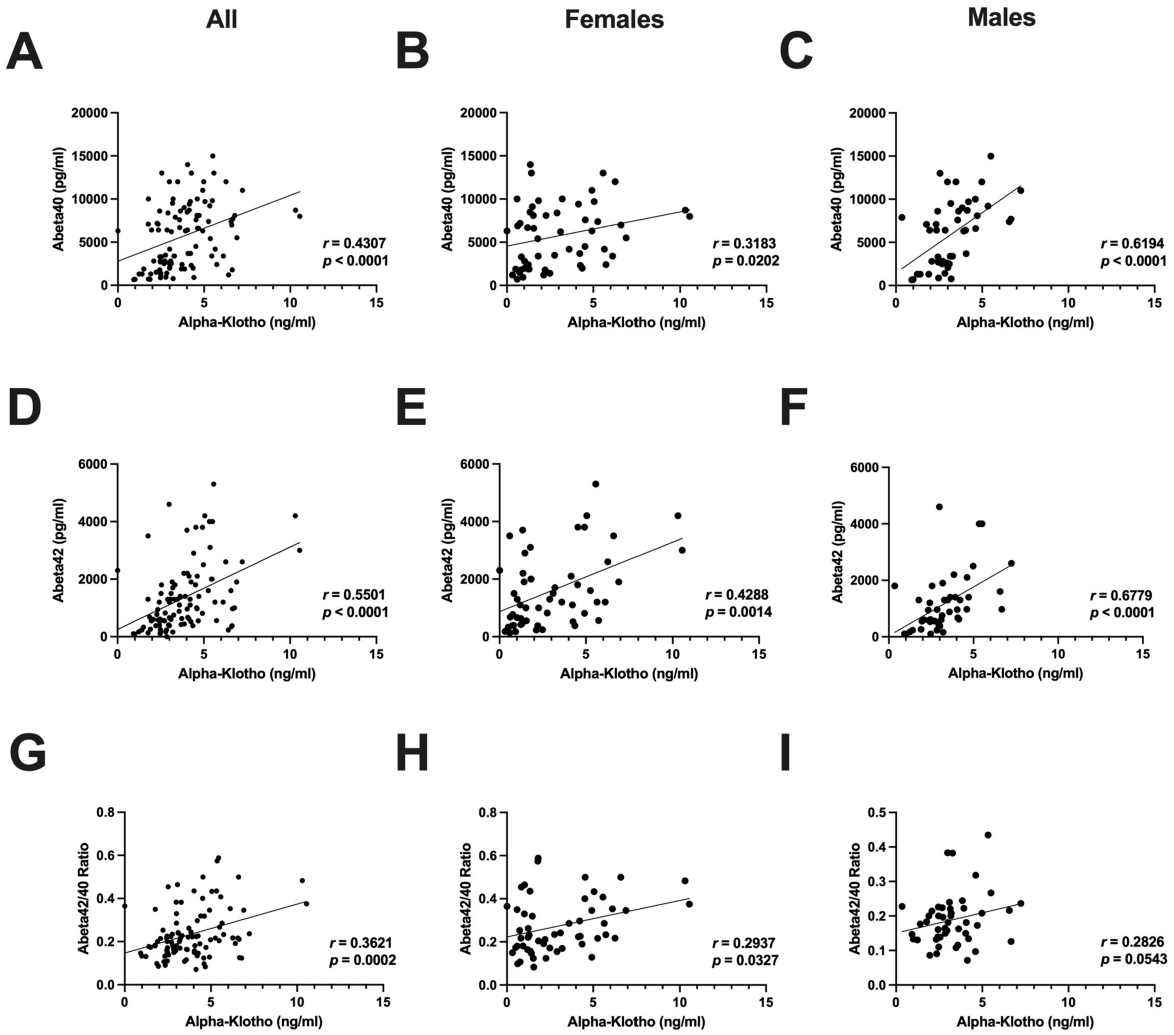
**(A)** CSF *α*-klotho levels correlated with CSF Aβ40 levels in all NHPs (*r* = 0.4307, *p* < 0.0001, Spearman). **(B)** CSF *α*-klotho levels correlated with CSF Aβ40 levels only in females (*r* = 0.3183, *p* = 0.0202, Spearman). **(C)** CSF *α*-klotho levels correlated with Aβ40 levels only in males (*r* = 0.6194, *p* < 0.001, Spearman). **(D)** CSF *α*-klotho levels correlated with CSF Aβ42 levels in both females and males (*r* = 0.5501, *p* < 0.0001, Spearman). **(E)** CSF *α*-klotho levels correlated with CSF Aβ42 levels in only females (*r* = 0.4288, *p* = 0.0014, Spearman). **(F)** CSF *α*-klotho levels correlated with CSF Aβ42 levels in only males (*r* = 0.6779, *p* < 0.0001, Spearman). **(G)** CSF *α*-klotho levels correlated with the CSF Aβ42/40 ratio in all NHPs (*r* = 0.3621, *p* = 0.0002, Spearman). **(H)** CSF *α*-klotho levels correlated with the CSF Aβ42/40 ratio only in females (*r* = 0.2937, *p* = 0.0327, Spearman). **(I)** There was a trend toward a correlation between CSF *α*-klotho and the CSF Aβ42/40 ratio only in males (*r* = 0.2826, *p* = 0.0543, Spearman). In all panels, data of animals are displayed regardless of treatment.

To determine whether the relationship between CSF *α*-klotho and Aβ42, and CSF *α*-klotho and the Aβ42/40 ratio, was limited to the HT study of female rhesus macaques. However, we also assessed these relationships in all of our other archival rhesus macaque CSF samples. In contrast to the HT study, CSF *α*-klotho levels correlated with Aβ40 levels in all NHPs (Figure 2A), in only females (Figure 2B), and in only males (Figure 2C), with a stronger correlation in males than females. CSF *α*-klotho levels also correlated with Aβ42 levels in all NHPs (Figure 2D), in only females (Figure 2E), and in only males (Figure 2F), with a stronger correlation in males than females. CSF *α*-klotho levels also correlated with the Aβ42/40 ratio in in all NHPs (Figure 2G) and in only females (Figure 2H), with a trend toward a correlation in only males (Figure 2I).

We next determined whether these relationships were also seen in serum. In contrast to CSF *α*-klotho levels, serum *α*-klotho levels did not correlate significantly with CSF Aβ40 (*r* = 0.01885, *p* = 0.9354, Spearman) nor with Aβ42 (*r* = 0.01884, *p* = 0.9354) levels, nor with the Aβ42/40 ratio (*r* = 0.1910, *p* = 0.4070).

**Figure 3:**
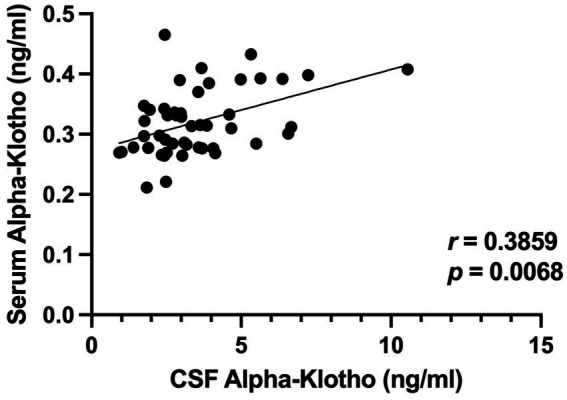
CSF and serum *α*-klotho levels were correlated (*r* = 0.3859, *p* = 0.0068, Spearman).

In our previous human study, we found CSF and serum *α*-klotho levels to be significantly correlated (Gupta et al., 2022). Therefore, we assessed whether this relationship is also evident in the NHP samples. As in humans, CSF and serum *α*-klotho levels in the NHPs were found to be positively correlated (*r* = 0.3859, *p* = 0.0068, Spearman), with higher *α*-klotho levels in CSF than serum (Figure 3).

### 3.3 Elderly humans without dementia (controls)

**Figure 4:**
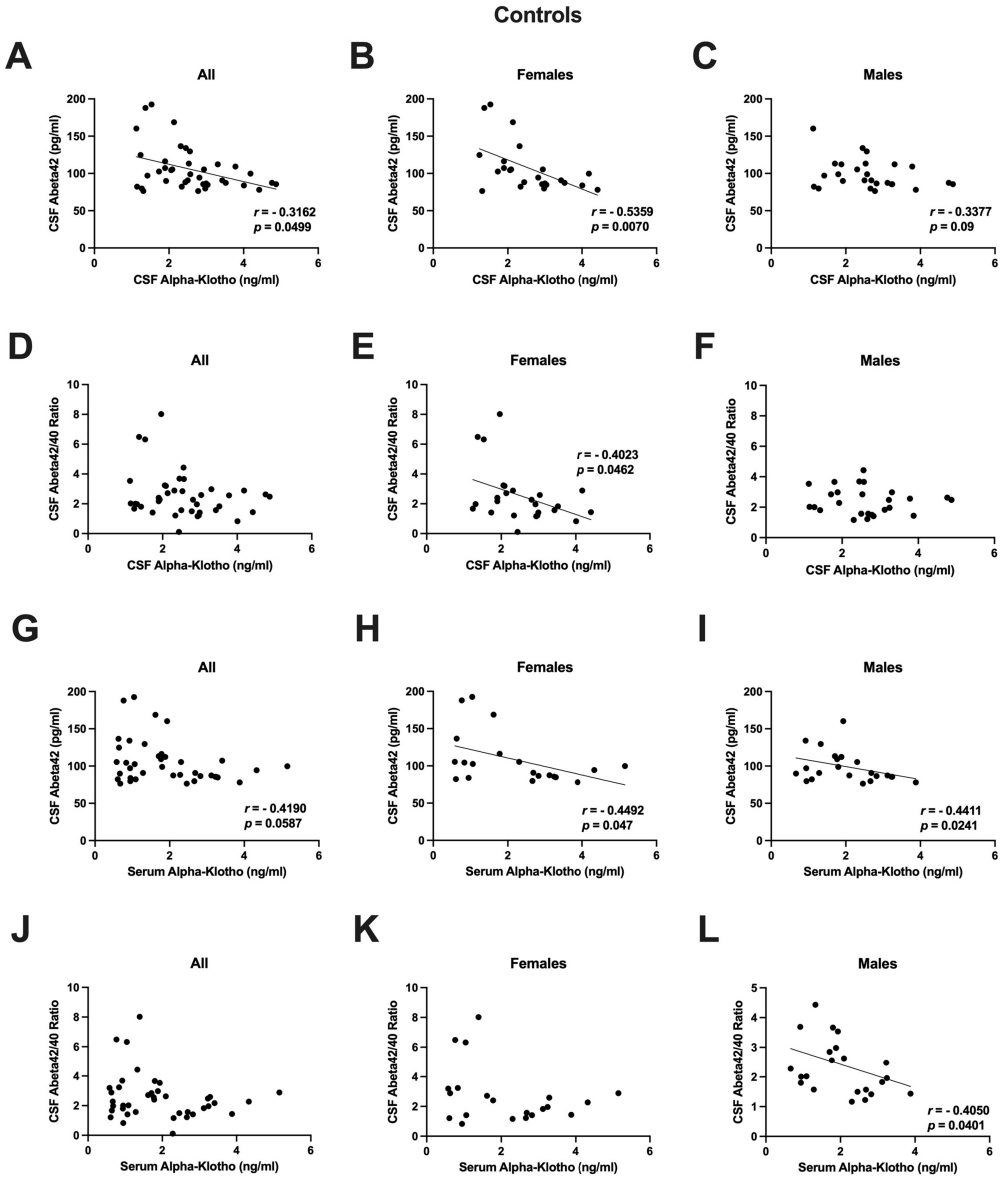
**(A)** There was a negative correlation between CSF *α*-klotho levels and Aβ42 levels in all controls (*r* = −0.3162, *p* = 0.0499, Spearman). **(B)** There was a negative correlation between CSF *α*-klotho levels and Aβ42 levels in only females (*r* = −0.5359, *p* = 0.0070, Spearman). **(C)** There was a trend toward a negative correlation between CSF *α*-klotho levels and Aβ42 levels in only males (*r* = −0.33377, *p* = 0.09, Spearman). **(D)** There was no correlation between CSF *α*-klotho levels and the Aβ42/40 ratio in all controls. **(E)** CSF *α*-klotho levels were negatively correlated with the Aβ42/40 ratio in females (*r* = −0.4023, *p* = 0.0462, Spearman). **(F)** There was no correlation between CSF *α*-klotho levels and the Aβ42/40 ratio in only males. **G.** There was a trend toward a negative correlation in all controls (*r* = −0.4190, *p* = 0.0587, Spearman). **(H)** Serum *α*-klotho levels correlated negatively with CSF Aβ42 levels in females (*r* = −0.4492, *p* = 0.047, Spearman). **(I)** Serum *α*-klotho levels correlated negatively with CSF Aβ42 levels in males (*r* = −0.4411, *p* = 0.0241, Spearman). **(J)** There was no correlation between serum *α*-klotho levels and the CSF Aβ42/40 ratio in all controls. **(K)** There was no correlation between serum *α*-klotho levels and the CSF Aβ42/40 ratio in only females. **(L)** Serum *α*-klotho levels correlated negatively with the CSF Ab42/40 ratio in only males (*r* = −0.4050, *p* = 0.0401, Spearman).

Next, we examined how CSF or serum *α*-klotho levels correlate with Aβ40, Aβ42, or the Aβ42/40 ratio in control humans (i.e., without dementia). There was no significant correlation between CSF *α*-klotho levels and Aβ40 levels in all controls, only females, or only males. However, there was a negative correlation between CSF *α*-klotho levels and Aβ42 levels in all controls (Figure 4A) and only females (Figure 4B), with a trend toward a correlation in only males (Figure 4C). CSF *α*-klotho levels were also negatively correlated with the Aβ42/40 ratio in females (Figure 4E), but no significant correlation was evident in all controls (Figure 4D) nor in only males (Figure 4F).

Although outside the brain, the main source of *α*-klotho is the kidneys and *α*-klotho cannot penetrate the blood–brain-barrier, there was a correlation between CSF and serum *α*-klotho levels in this cohort (Kundu et al., 2022) and peripheral administration of an *α*-klotho fragment enhanced cognition in mice (Leon et al., 2017). Therefore, we also assessed whether serum *α*-klotho levels are correlated with CSF Aβ measures. Serum *α*-klotho levels did not correlate with CSF Aβ40 levels in all, female, nor male controls. However, serum *α*-klotho levels correlated negatively with CSF Aβ42 levels in females (Figure 4H) and males (Figure 4I), and there was a trend toward a negative correlation in all controls (Figure 4G). Serum *α*-klotho levels also correlated negatively with the CSF Aβ42/40 ratio in only males (Figure 4L), but no correlation was seen in all (Figure 4J) nor in only females (Figure 4K).

### 3.4 Patients with dementia

**Figure 5:**
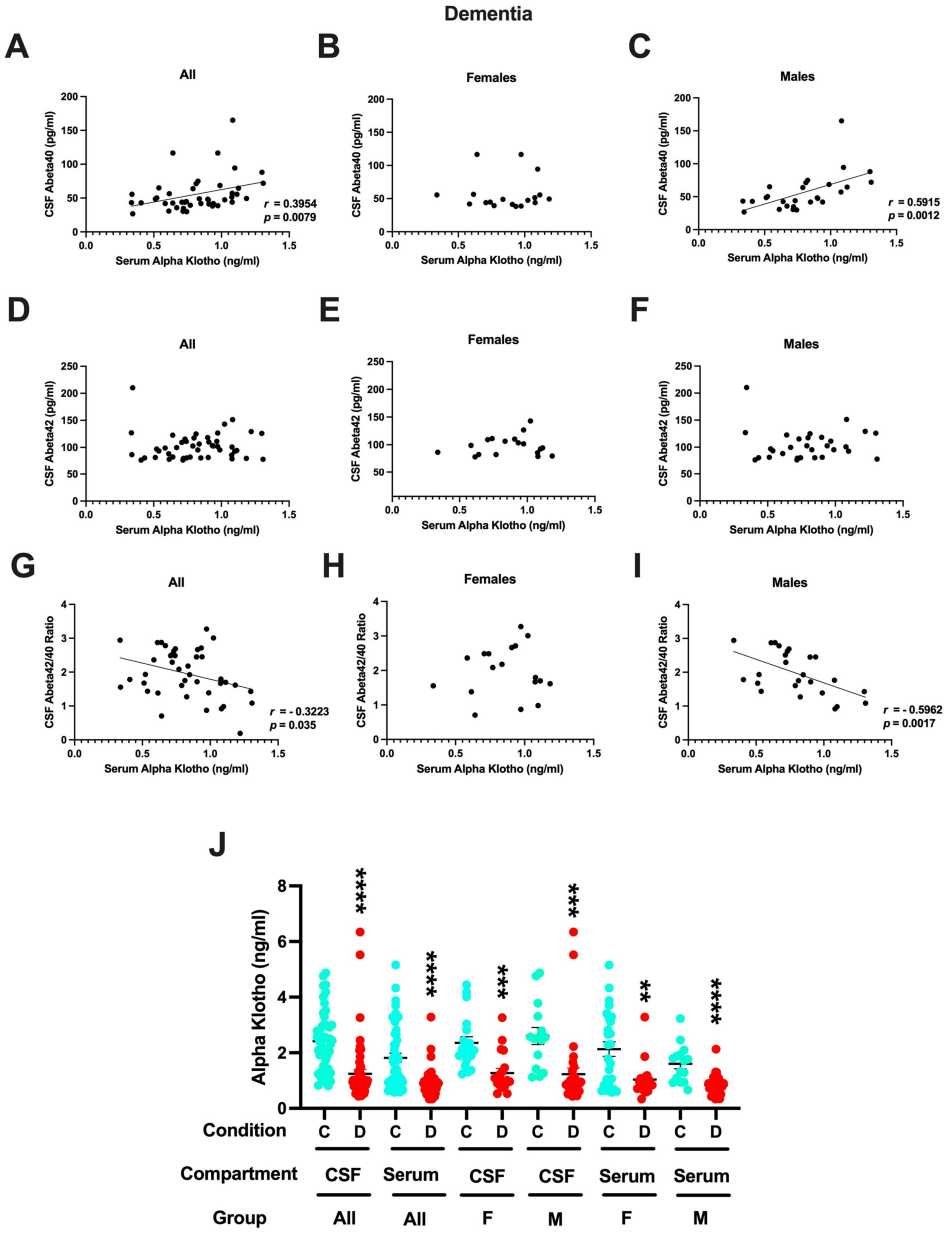
**(A)** Serum *α*-klotho levels did correlate positively with CSF Aβ40 levels in all patients with dementia (*r* = 0.3954, *p* = 0.0079, Spearman). **(B)** No relationship between serum *α*-klotho levels and CSF Aβ40 levels was seen in females. **(C)** Serum *α*-klotho levels did correlate positively with CSF Aβ40 levels in males with dementia (*r* = 0.5915, *p* = 0.0012, Spearman). **(D)** Serum *α*-klotho levels did not correlate with CSF Aβ42 levels in all patients with dementia. **(E)** Serum *α*-klotho levels did not correlate with CSF Aβ42 levels in female patients with dementia. **(F)** Serum *α*-klotho levels did not correlate with CSF Aβ42 levels in female patients with dementia. **(G)** Serum *α*-klotho levels correlated negatively with the CSF Aβ42/40 ratio in all patients with dementia (*r* = −0.3223, *p* = 0.035, Spearman). **(H)** No relationship between serum *α*-klotho levels and the CSF Aβ42/40 ratio was seen in females with dementia. **(I)** Serum *α*-klotho levels correlated negatively with the CSF Aβ42/40 ratio in male patients with dementia (*r* = −0.5962, *p* = 0.0017, Spearman). **(J)** In females and males combined and females and males separately, the CSF and serum *α*-klotho levels were much higher in controls than in those with dementia. CSF all: *t* = 5.456, ****p < 0.0001; serum all: *t* = 5.277, ****p < 0.0001; CSF females only: *t* = 2.358, ***p = 0.0003; CSF males: *t* = 3.566, ***p = 0.0008; serum females only: *t* = 3.296, **p = 0.0020; serum males only: *t* = 5.215, ****p < 0.0001.

In contrast to controls, in patients with dementia, CSF *α*-klotho levels did not correlate significantly with CSF Aβ42 levels or the Aβ42/40 ratio. However, serum *α*-klotho levels did correlate positively with CSF Aβ40 levels in all patients with dementia (Figure 5A) and in only males with dementia (Figure 5C). No relationship between serum *α*-klotho levels and CSF Aβ40 levels was seen in females.

Also, in contrast to controls, serum *α*-klotho levels did not correlate with CSF Aβ42 levels either in all (Figure 5D), only females (Figure 5E), nor only in males (Figure 5F). Serum *α*-klotho levels correlated negatively with the CSF Aβ42/40 ratio in all patients with dementia (Figure 5G) and in only male patients with dementia (Figure 5I). No relationship between serum *α*-klotho levels and the CSF Aβ42/40 ratio was seen in females (Figure 5H).

In the current study, we did not see a difference in CSF Aβ42 levels between controls (105.5 ± 4.7 pg./mL) and patients with dementia (102.5 ± 3.6 pg./mL).

Finally, we compared the CSF and serum *α*-klotho levels in controls and dementia patients. In females and males combined and separately, the CSF and serum *α*-klotho levels were at least twice as high in controls than in those with dementia (Figure 5J).

## 4 Discussion

In NHPs, there was an effect of HT on CSF *α*-klotho levels. Ovariectomized animals that were maintained for ~30 months on a WSD and received immediate E2 hormone therapy (HT), had higher *α*-klotho levels compared to ovary-intact controls. However, in untreated OVX animals CSF *α*-klotho levels were not lower than in ovary-intact controls. Notably, the inflammatory potential of a person’s diet (Tabung et al., 2015), is associated with low *α*-klotho serum levels (Zhang et al., 2022). More specifically, pro-inflammatory cytokines like tumor necrosis factor *α*, interferon *γ*, and interleukin 6 all reduce *α*-klotho gene expression in the kidneys (Izquierdo et al., 2012), the main source of *α*-klotho in the blood, while resveratrol induces *α*-klotho expression in the kidneys (Hsu et al., 2014) and a high intake of nuts is associated with elevated *α*-klotho levels in blood (Jurado-Fasoli et al., 2019). Therefore, the effects of HT on CSF *α*-klotho levels might be more pronounced under a WSD than a standard diet. Consistent with this notion, there was no increase in CSF *α*-klotho levels in old NHP males who received testosterone and DHEA for 6 months (1.246 ± 0.310 ng/mL; controls: 1.114 ± 0.220 ng/mL). As in the human and NHP data, *α*-klotho levels in the serum and CSF were correlated, HT would be expected to increase CSF *α*-klotho levels in humans as well. However, the species-dependent relationships between *α*-klotho and Aβ and the higher levels of both in the CSF of NHPs compared to humans indicate that it is important to be cautious about extrapolating from results in NHPs to clinical practice in humans.

CSF and serum *α*-klotho levels in NHPs were correlated, with higher levels in CSF than serum. The pattern with higher *α*-klotho levels in CSF than serum was also seen in some (Gaitan et al., 2022) but not all (Gupta et al., 2022) human studies.

In CSF, *α*-klotho levels were about 50% lower in patients with dementia than in controls. The levels seen in the dementia patients were comparable to those seen in AD dementia patients in an independent study (1.1 ng/mL) (Grøntvedt et al., 2022). However, the *α*-klotho levels in the CSF of the controls in that study were 1.2–1.3 ng/mL and lower than the CSF *α*-klotho levels seen in the current study. Because controls showed an age-related decrease in CSF *α*-klotho levels, it is conceivable that differences in the percentage of younger control participants in the two studies might have contributed to these different CSF *α*-klotho levels. With regards to the dementia patients in this cohort, only a minority of subjects had relatively advanced dementia at the time of CSF sampling - i.e., only about 4 (see Figure 2 in Gupta et al., 2022).

While CSF *α*-klotho levels were positively correlated with CSF Aβ40, Aβ42, and the Aβ42/40 ratio in female and male NHPs, CSF *α*-klotho levels were only correlated with CSF Aβ42 and the Aβ42/40 ratio in control women and with CSF Aβ42 in all controls (for a summary of the correlations, see Table 1). No relationship between CSF *α*-klotho levels and CSF Aβ measures were seen in demented patients. CSF Aβ measures are often interpreted reflecting beneficial Aβ clearance from the brain (Li et al., 2022; Elbert et al., 2022).

**Table 1:** Summary of significant correlations between *α*-klotho levels and Aβ40, Aβ42, and the Aβ42/40 ratio.^1^

	All NHPs	Female NHPs	Male NHPs
CSF *α*-klotho; CSF Aβ40	Positive	Positive	Positive
CSF *α*-klotho; CSF Aβ42	Positive	Positive	Positive
CSF *α*-klotho; CSF Aβ42/40 ratio	Positive	Positive	Positive

	All controls	Female controls	Male controls
CSF *α*-klotho; CSF Aβ42	Negative	Negative	
CSF *α*-klotho; CSF Aβ42/40 ratio		Negative	
Serum *α*-klotho; CSF Aβ42		Negative	Negative
Serum *α*-klotho; CSF Aβ42/40 ratio			Negative

	All dementia	Female dementia	Male dementia
Serum *α*-klotho; CSF Aβ40	Positive		Positive
Serum *α*-klotho; CSF Aβ42/40 ratio	Negative		Negative

^1^Only significant correlations are indicated. The positive correlations are indicated in orange, the negative correlations in blue.

Next, we compared the relative levels of these biomarkers in humans and NHPs (Table 2) to explore whether they might be able to explain the relative protections of NHPs to develop full-blown neurodegeneration as seen in AD.

**Table 2:** Comparison of relative CSF and serum *α*-klotho levels and relative CSF apoE, Aβ40, Aβ42 levels in humans and NHPs.^1^

Measure	Relative levels	Fold change
CSF *α*-klotho	NHPs > Humans	1.7
Serum *α*-klotho	NHPs < Humans	12
CSF apoE	NHPs > Humans	1.6
CSF Aβ42	NHPs > Humans	26.7
CSF Aβ40	NHPs > Humans	90.9

^1^The measures for which the levels were higher in NHPs than humans and the fold change are indicated in orange. The measures for which the levels were lower in NHPs than human and the fold change are indicated in blue.

The *α*-klotho levels in the CSF were lower in AD dementia patients than controls and lower in controls than in NHPs; the range of the *α*-klotho levels in the CSF was <6 ng/mL in controls and patients with dementia and <10 ng/mL in NHPs - i.e., a 1.7-fold difference. It is plausible that the higher CSF *α*-klotho levels in NHPs than in humans might provide protection against developing an AD-like condition in the former. In contrast to CSF, serum *α*-klotho levels were lower in serum of NHPs (< 0.5 ng/mL) than humans (< 6 ng/mL) - i.e., a 12-fold difference.

The apoE levels in the CSF were also higher in NHPs (< 280 ng/mL) than humans [< 175 ng/mL (Gupta et al., 2022)] - i.e., a 1.6-fold difference.

Compared to CSF *α*-klotho and CSF apoE levels, a much more profound species difference was seen in CSF Aβ40 and Aβ42 levels in humans versus NHPs. In human controls and AD dementia patients, CSF Aβ42 levels were <210 pg/mL; in contrast CSF Aβ42 levels in NHPs were <5,600 pg/mL - i.e., a 26.7-fold difference. In human controls and AD dementia patients, CSF Aβ40 levels were <165 pg/mL; in contrast CSF Aβ40 levels in NHPs were <15,000 pg/mL - i.e., a 90.9-fold difference.

In another study analyzing Aβ levels in the CSF of Rhesus macaques, the CSF Aβ40 levels were <6,000 pg./mL, while the CSF Aβ42 levels were <1,200 pg./mL (Robertson et al., 2022) - i.e., 2.5-fold and 4.7-fold lower, respectively than in our study. Regardless of this difference between studies, there appears to be a marked species difference with much higher CSF Aβ40 levels and CSF Aβ42 levels in NHPs than in humans.

Clearance of Aβ42 and Aβ40 into the CSF is thought to be critical for protection against developing AD (Elbert et al., 2022). In NHPs, the relatively much higher levels of Aβ40 and Aβ42 in the CSF might contribute in their protection from developing an AD-like condition. As *α*-klotho levels in the choroid plexus decline with age and increasing them enhances Aβ clearance, the higher CSF levels of *α*-klotho in NHPs than in humans are likely beneficial (Haley et al., 2010). Higher CSF levels of apoE might be beneficial for clearance in AD as well. In women, baseline CSF apoE levels are associated with baseline Aβ and total tau levels as well as longitudinal changes in these biomarkers, while in men baseline apoE levels were associated with phosphorylated and total tau levels (Liu et al., 2021).

Although CSF and serum *α*-klotho levels are distinct pools and *α*-klotho cannot pass the blood–brain-barrier (Landry et al., 2021), human serum *α*-klotho levels were also correlated with CSF Aβ measures. Serum *α*-klotho levels were positively correlated with CSF Aβ40 but negatively with the CSF Aβ42/40 ratio in male patients with dementia and similarly correlated in all patients with dementia. However, while serum *α*-klotho levels correlated negatively with CSF Aβ42 levels in female and male controls, serum *α*-klotho levels correlated positively with CSF Aβ40 levels in males and in all patients with dementia. Most dementia patients in this study were diagnosed with probable or possible AD and the CSF Aβ42/40 ratio is superior than CSF Aβ42 in differentiating AD pathology from non-AD pathology (Costantinides et al., 2023). Therefore, the negative relationship between serum *α*-klotho levels and the CSF Aβ42/40 ratio seen in male controls and males and all with dementia is likely especially clinically relevant. We recognize that the CSF and serum contain different proteins and nucleic acids and as a result they might affect the measurements of *α*-klotho, apoE, Aβ40, and Aβ42 in these two distinct fluids. In addition, since *α*-klotho cannot pass the blood–brain-barrier, differences in regulatory mechanisms of *α*-klotho inside and outside the brain could influence the interpretation of serum *α*-klotho as a biomarker of amyloid pathology within the brain.

Limitations of the current study include the sample size of the number of humans and NHPs, the heterogeneity of dementia diagnoses, including probable versus possible AD. It will be important to expand the human data to other neurodegenerative conditions to assess whether the pattern seen in AD is unique or not. Furthermore, although standard curves were used for the analyses of all measures, the NHP Aβ assay involves Luminex technology and magnetic microparticles (beads) that are not present in the human Aβ40 and Aβ42 ELISAs. Therefore, we cannot exclude that potential differences in these two assays might have contributed to the species differences in the Aβ40 and Aβ42 levels in CSF of humans and NHPs. For the *α*-klotho levels, the same ELISAs were used to analyze the human and NHP samples.

The directional difference in the correlations between CSF *α*-klotho levels and Aβ measures in NHPs and human controls might be due to distinct environmental factors and a less heterogenous genetic background in NHPs than humans (Rogers and Gibbs, 2014). However, the CSF levels of Aβ40, Aβ42, *α*-klotho, and apoE were all higher in NHPs than humans and the species difference in the levels of these markers might also contribute to the observed directional difference in correlations. We recognize that it is also conceivable that correlations between *α*-klotho and Aβ might not be conserved between humans and NHPs. As higher CSF and serum levels of *α*-klotho were seen in female and male controls than sex-matched patients with dementia, the NHP and human results suggest that the beneficial effects of *α*-klotho on CSF Aβ physiology involve species-dependent mechanisms. Future studies are warranted to understand these mechanisms and evaluate novel therapeutic strategies to increase serum and CSF *α*-klotho levels in cognitively healthy and demented elderly humans.

## Data Availability

The original contributions presented in the study are included in the article/supplementary material, further inquiries can be directed to the corresponding author.
